# Value of Vitamin D Metabolite Ratios in 3 Patients as Diagnostic Criteria to Assess Vitamin D Status

**DOI:** 10.1210/jcemcr/luae095

**Published:** 2024-06-28

**Authors:** Zhinous Shahidzadeh Yazdi, Elizabeth A Streeten, Hilary B Whitlatch, Salma A Bargal, Amber L Beitelshees, Simeon I Taylor

**Affiliations:** Department of Medicine, Division of Endocrinology, Diabetes, and Nutrition, University of Maryland School of Medicine, Baltimore, MD 21201, USA; Department of Medicine, Division of Endocrinology, Diabetes, and Nutrition, University of Maryland School of Medicine, Baltimore, MD 21201, USA; Department of Medicine, Division of Endocrinology, Diabetes, and Nutrition, University of Maryland School of Medicine, Baltimore, MD 21201, USA; Department of Medicine, Division of Endocrinology, Diabetes, and Nutrition, University of Maryland School of Medicine, Baltimore, MD 21201, USA; Department of Medicine, Division of Endocrinology, Diabetes, and Nutrition, University of Maryland School of Medicine, Baltimore, MD 21201, USA; Department of Medicine, Division of Endocrinology, Diabetes, and Nutrition, University of Maryland School of Medicine, Baltimore, MD 21201, USA

**Keywords:** VitD status, VitD metabolite ratios, 24, 1,25-dihydroxyvitamin D, 25-hydroxyvitamin D, 24-hydroxylase, PTH

## Abstract

Although clinical guidelines recommend measuring total plasma 25-hydroxyvitamin D (25[OH]D) to assess vitamin D (VitD) status, this index does not account for 3-fold inter-individual variation in VitD binding protein (VDBP) level. We present 3 individuals with total plasma 25(OH)D levels of 10.8 to 12.3 ng/mL (27-30.7 nmol/L). Because Endocrine Society guidelines define VitD deficiency as 25(OH)D ≤ 20 ng/mL (50 nmol/L), all 3 would be judged to be VitD deficient. VitD3 supplementation increased 25(OH)D to the range of 31.7 to 33.8 ng/mL (79.1-84.4 nmol/L). Patient #1 exhibited secondary hyperparathyroidism; VitD3 supplementation decreased parathyroid hormone (PTH) by 34% without a clinically significant change in PTH levels in the other 2 individuals. Thus, 25(OH)D level did not distinguish between the 1 patient who had secondary hyperparathyroidism and the 2 who did not. We therefore inquired whether VitD metabolite ratios (which are VDBP-independent) might distinguish among these 3 individuals. Of all the assessed ratios, the 1,25(OH)_2_D/24,25(OH)_2_D ratio was the most informative, which had a value of 102 pg/ng in the individual with secondary hyperparathyroidism but lower values (41 and 20 pg/ng) in the other 2 individuals. These cases illustrate the value of the 1,25(OH)_2_D/24,25(OH)_2_D ratio to provide clinically relevant information about VitD status.

## Introduction

The Endocrine Society and The Institute of Medicine suggest measuring total plasma 25-hydroxyvitamin D (25[OH]D) to assess vitamin D (VitD) status but suggest different definitions of VitD deficiency and sufficiency [1, 2]. Recent publications question the value of VitD in preventing bone fracture in the general population [3]. The existing controversy in the literature is due in part to the fact that total 25(OH)D measurement does not account for the 3-fold inter-individual variation in VitD binding protein (VDBP) levels [4]. Although > 99% of circulating VitD metabolites are bound to proteins (albumin and VDBP), the biologically relevant form is the free fraction [5]. Two enzymes play critical roles in VitD metabolism. 1α-Hydroxylase converts 25(OH)D into 1,25-dihydroxyvitamin D (1,25[OH]_2_D)—the biologically active form. 24-Hydroxylase mediates metabolic clearance of 25(OH)D and 1,25(OH)_2_D by converting them to 24,25-dihydroxyvitamin D (24,25[OH]_2_D) and 1,24,25-trihydroxyvitamin D (1,24,25[OH]_3_D), respectively [6, 7]. Using ratios of VitD metabolites provides an indirect approach to account for VDBP levels [8‐10]. Tang et al suggested that the 1,25(OH)_2_D/24,25(OH)_2_D ratio is superior to total 25(OH)D to assess VitD status [10]. This approach provides VDBP-independent indices that assess VitD status more accurately and can better predict clinical outcomes. Other VitD metabolite ratios have been suggested by others [8, 11, 12]. We conducted a clinical trial in otherwise healthy individuals with 25(OH)D ≤ 20 ng/mL (≤ 50 nmol/L) studied before and after VitD3 supplementation [13]. This study identified 3 individuals with total plasma 25(OH)D of 10.8 to 12.3 ng/mL (27-30.7 nmol/L), whom the Endocrine Society guidelines classify as VitD deficient and for whom they recommend VitD supplementation [1]. When we assessed responses to VitD3 supplementation, one patient had what we propose to define as “overt VitD deficiency” in association with secondary hyperparathyroidism. One patient appeared to be VitD sufficient, and one appeared to have what we propose to label as “subclinical VitD deficiency” without secondary hyperparathyroidism. These cases illustrate the potential utility of VitD metabolite ratios in assessing clinical VitD status.

## Case Presentations

All blood samples were obtained at ∼7 Am in the fasting state. The reference ranges for all lab values are listed in the tables.

***Patient #1*** was a 43-year-old otherwise healthy Amish man with total 25(OH)D = 11.7 ng/mL (29 nmol/L). He did not report major illnesses nor a history of bone fractures or osteoporosis in himself, his parents, or any of his 8 siblings. He did not take prescribed medications or over-the-counter supplements. Relevant baseline lab results include: 1,25(OH)_2_D = 51 pg/mL (127 pmol/L); 24,25(OH)_2_D = 0.5 ng/mL (1.2 nmol/L); intact parathyroid hormone (iPTH) = 62 pg/mL (6.6 pmol/L); Ca = 9.2 mg/dL (2.3 mmol/L); P = 3.1 mg/dL (1 mmol/L); and albumin = 4.2 g/dL.

***Patient #2*** was a 65-year-old otherwise healthy Amish man with 25(OH)D = 10.8 ng/mL; 27 nmol/L. He did not report medical illnesses nor a history of bone fractures or osteoporosis. Both parents died from strokes. He reported hip fracture in his father following a fall. He did not use prescribed medications but took 2 over-the-counter dietary supplements, neither of which contained VitD. Relevant baseline lab results include: 1,25(OH)_2_D = 33 pg/mL (82 pmol/L); 24,25(OH)_2_D = 0.8 ng/mL (2 nmol/L); iPTH = 29 pg/mL (3.1 pmol/L); Ca = 9.3 mg/dL (2.3 mmol/L); P = 3.3 mg/dL (1.1 mmol/L); and albumin = 4.1 g/dL.

***Patient #3*** was a 37-year-old otherwise healthy Amish man with 25(OH)D = 12.3 ng/mL (30.7 nmol/L). He did not report medical illnesses nor a history of bone fractures or osteoporosis in himself, his parents, or any of his 10 siblings. He was not on any prescribed medication but took 3 dietary supplements none of which contained VitD. Relevant baseline lab results include: 1,25(OH)_2_D = 19 pg/mL (47.4 pmol/L); 24,25(OH)_2_D = 0.9 ng/mL (2.3 nmol/L); iPTH = 51 pg/mL (5.4 pmol/L); Ca = 9.1 mg/dL (2.3 mmol/L); P = 4.2 mg/dL (1.4 mmol/L); and albumin = 4.4 g/dL.

Table 1 summarizes demographic and anthropometric characteristics. Table 2 summarizes lab results both before (baseline) and after VitD3 supplementation for all 3 patients.

**Table 1.** The demographic and anthropometric characteristics

	Patient #1	Patient #2	Patient #3	Reference ranges when applicable
Age (years)	43	65	37	
Sex	Male	Male	Male	
Weight (kg)	74.2	90.2	70.6	
Height (cm)	170.7	162.5	181.7	
BMI (kg/m^2^)	25.7	34	21.4	
eGFR (mL/min/1.73 m^2^)	113	92	100	≥60
Creatinine	0.76 mg/dL	0.85 mg/dL	0.97 mg/dL	0.60-1.35 mg/dL
	(67.2 umol/L)	(75.2 umol/L)	(85.8 umol/L)	(53.1-119.4 umol/L)
Fasting blood sugar	131 mg/dL	84 mg/dL	84 mg/dL	70-100 mg/dL
	(7.3 mmol/L)	(4.7 mmol/L)	(4.7 mmol/L)	(3.9-5.6 mmol/L)
HbA1c (%)	5.1	5.6	5.3	<5.7%
**TSH** (mIU/L)	1.87	2.4	2.4	
Albumin (g/dL)	4.2	4.1	4.4	3.6-5.1 g/dL

Abbreviations: BMI, body mass index; eGFR, estimated glomerular filtration rate; HbA1c, glycated hemoglobin; TSH, thyrotropin (thyroid stimulating hormone).

**Table 2.** Bone related parameters before and after VitD3 supplementation.

	Patient #1	Patient #2	Patient #3	Reference ranges
**25(OH)D**				*^a^* Deficiency: ≤ 20 (≤ 50 nmol/L)
Before VitD	11.7 ng/mL (29 nmol/L)	10.8 ng/mL (27 nmol/L)	12.3 (30.7 nmol/L)	Insufficiency: 21-29 (52-72 nmol/L)
After VitD	31.7 (79.1 nmol/L)	31.8 (79.4 nmol/L)	33.8 (84.4 nmol/L)	Sufficiency: ≥ 30 (75 nmol/L)
**1,25(OH)_2_D**				
Before VitD	51 pg/mL (127 pmol/L)	33 pg/mL (82 pmol/L)	19 pg/mL (47 pmol/L)	Adults:
After VitD	34 (85 pmol/L)	29 (72 pmol/L)	22 (55 pmol/L)	18-72 pg/mL (45-180 pmol/L)
**24,25(OH)_2_D**				
Before VitD	0.5 ng/mL (1.3 nmol/L)	0.8 ng/mL (2 nmol/L)	0.9 ng/mL (2.3 nmol/L)	–
After VitD	2.9 ng/mL (7.2 nmol/L)	2.8 ng/mL (7 nmol/L)	4.1 ng/mL (10.2 nmol/L)	
**Calcium**				
Before VitD	9.3 mg/dL (2.32 mmol/L)	9.6 mg/dL (2.395 mmol/L)	9.7 mg/dL (2.4 mmol/L)	Males ≥ 20 yrs:
After VitD	9.1 (2.27 mmol/L)	9.4 mg/dL (2.345 mmol/L)	9.7 mg/dL (2.4 mmol/L)	8.6-10.3 mg/dL (2.2-2.6 mmol/L)
**Phosphorus**				
Before VitD	3.1 (1 mmol/L)	3.3 (1.1 mmol/L)	4.2 (1.36 mmol/L)	2.5-4.5 mg/dL (0.8-1.5 mmol/L)
After VitD	3.2 (1 mmol/L)	4 (1.3 mmol/L)	4.4 (1.42 mmol/L)	
**iPTH**				
Before VitD	62 pg/mL (6.6 pmol/L)	29 pg/mL (3.1 pmol/L)	51 pg/mL (5.4 pmol/L)	≥19 yrs:
After VitD	41 pg/mL (4.4 pmol/L)	30 pg/mL (3.2 pmol/L)	54 pg/mL (5.7 pmol/L)	14-64 pg/mL (1.5-6.8 pmol/L)
**iFGF23**				
Before VitD	50.4 pg/mL)	59.9 pg/mL)	57.1 pg/mL)	18.6-59.8 pg/mL
After VitD	60.5 pg/mL)	67.6 pg/mL)	62.6 pg/mL)	
**ßCTX**				Males: 30-39 yrs (70-780 pg/mL)
Before VitD	425 pg/mL	189 pg/mL	365 pg/mL	40-49 yrs (60-700 pg/mL)
After VitD	578 pg/mL	182 pg/mL	317 pg/mL	50-68 yrs (87-345 pg/mL)
**P1NP**				Males:
Before VitD	32 mcg/L	23 mcg/L	40 mcg/L	23-60 yrs (30-110 mcg/L)
After VitD	31 mcg/L	25 mcg/L	35 mcg/L	> 60 yrs (not stablished)

Abbreviations: 1,25(OH)2D, 1,25-dihydroxyvitamin D; 24,25(OH)2D, 24,25-dihydroxyvitamin D; 25(OH)D, 25-hydroxyvitamin D; βCTX, type I collagen C-terminal telopeptide; iFGF23, fibroblast growth factor 23; iPTH, intact parathyroid hormone; P1NP, procollagen type 1 amino-terminal propeptide-1; PTH, parathyroid hormone; VitD, vitamin D.

^***a***^The Endocrine Society's proposed cutoffs to define VitD status.

## Diagnostic Assessment

Although all 3 patients met Endocrine Society's criteria for VitD deficiency [1], only one had “overt VitD deficiency” in association with secondary hyperparathyroidism (as defined in the “Introduction” to this Case Report). One was VitD sufficient, and one had “subclinical” VitD insufficiency without secondary hyperparathyroidism (Table 3). Although VitD3 supplements decreased PTH levels by 34% in patient #1, patients #2 and #3 experienced only clinically insignificant 3% to 5% increases in PTH after VitD3 supplementation—indicating the absence of secondary hyperparathyroidism at baseline. Total plasma 25(OH)D has limitations in assessing VitD status as it does not account for VDBP levels. Two individuals with the same total 25(OH)D level may have different free fractions depending on their VDBP levels. The use of VitD metabolite ratios provides a VDBP-independent index of VitD status [9, 14]. For example, Tang et al [10] proposed the 1,25(OH)_2_D/24,25(OH)_2_D ratio to evaluate VitD status. They proposed that 1,25(OH)_2_D/24,25(OH)_2_D ratios ≥ 51 and < 35 pg/ng indicate VitD deficiency and sufficiency, respectively. They also suggested that the 1,25(OH)_2_D/24,25(OH)_2_D ratio > 100 is associated with increased risk of secondary hyperparathyroidism [10]. The 1,25(OH)_2_D/24,25(OH)_2_D ratio in patient #1 was 102 pg/ng—suggesting the diagnosis of VitD deficiency-induced secondary hyperparathyroidism. Patient #2 who exhibited the lowest 25(OH)D (10.8 ng/mL; 27 nmol/L) had a 1,25(OH)_2_D/24,25(OH)_2_D ratio of 41 pg/ng, consistent with VitD insufficiency. Patient #3 had a 1,25(OH)_2_D/24,25(OH)_2_D ratio of 20 pg/ng suggesting VitD sufficiency (Table 3).

**Table 3.** Three research participants exemplifying impact of precision diagnostics strategy for diagnosis of VitD deficiency

Patient	#1	#2	#3
**Baseline 25(OH)D**	11.7 ng/mL (29 nmol/L)	10.8 ng/mL (27 nmol/L)	12.3 ng/mL (30.7 nmol/L)
**Baseline 24,25(OH)_2_D**	0.5 ng/mL (1.3 nmol/L)	0.8 ng/mL (2 nmol/L)	0.9 ng/mL (2.3 nmol/L)
**Baseline 1,25(OH)_2_D**	51 pg/mL (127 pmol/L)	33 pg/mL (82 pmol/L)	19 pg/mL (47 pmol/L)
**Baseline iPTH**	62 pg/mL (6.6 pmol/L)	29 pg/mL (3.1 pmol/L)	51 pg/mL (5.4 pmol/L)
**Baseline iFGF23**	50 pg/mL	60 pg/mL	57 pg/mL
**[1,25(OH)_2_D]/[24,25(OH)_2_D]**	110 pg/ng	41 pg/ng	20 pg/ng
**[25(OH)D]/[1,25(OH)_2_D]**	0.23 ng/pg	0.33 ng/pg	0.65 ng/pg
**[24,25(OH)_2_D]/[25(OH)D]**	0.040	0.075	0.076
**PTH: response to VitD3** (Δ%)	−34%	+3%	+5%
**Patients’ VitD status classification based on The Endocrine Society's guideline**	VitD deficient	VitD deficient	VitD deficient
**Patients’ VitD status classification using [1,25(OH)_2_D]/[24,25(OH)_2_D] ratio**	Overt VitD deficiency	Subclinical VitD deficiency	VitD Sufficiency
**% Maximal 24-hydroxylase activity**	15%	18%	37%

This Table summarizes selected laboratory data and calculated VitD metabolite ratios for participant #1—the participant exhibiting the highest level of the 1,25(OH)_2_D/24,25(OH)_2_D ratio observed in our clinical trial [15]. Participants #2 and #3 were selected as matches for participant #1 based on having similar baseline total levels of plasma 25(OH)D.

Abbreviations: 1,25(OH)2D, 1,25-dihydroxyvitamin D; 24,25(OH)2D, 24,25-dihydroxyvitamin D; 25(OH)D, 25-hydroxyvitamin D; iFGF23, fibroblast growth factor 23; iPTH, intact parathyroid hormone; PTH, parathyroid hormone; VitD, vitamin D.

We developed a mathematical model to estimate relative activity of the 24-hydroxylase enzyme, which catabolizes 25(OH)D and 1,25(OH)_2_D [15]. Our model suggests that suppression of 24-hydroxylase activity provides the body's first line of defense to compensate for limited availability of VitD, resulting in slower metabolic clearance of 1,25(OH)_2_D and 25(OH)D. Secondary hyperparathyroidism, which develops in more severe VitD deficiency, leads to increased production of 1,25(OH)_2_D—the biologically active metabolite. The 1,25(OH)_2_D/24,25(OH)_2_D ratio incorporates the 24,25(OH)_2_D level in the denominator—thereby providing information about 24-hydroxylase activity. Our model estimated a ∼10-fold dynamic range for 24-hydroxylase activity as a function of 25(OH)D levels. Maximum and minimum suppression of 24-hydroxylase occurs when 25(OH)D is < 10 to 20 ng/mL (< 25-50 nmol/L) or > 50 ng/mL (125 nmol/L), respectively. Our mathematical model estimated that Case #1 (who had secondary hyperparathyroidism) exhibited only 15% of maximal 24-hydroxylase activity. Case #2, whom we classified as VitD insufficient, and case #3, whom we classified as VitD sufficient, exhibited 18% and 37% of maximal 24-hydroxylase activity, respectively. This mechanistic interpretation provides a physiological rationale for the diagnostic cutoffs proposed by Tang et al [10]. Even in 3 individuals with total 25(OH)D levels in the narrow range of 10.8 to 12.3 ng/mL (27-30.7 nmol/L), the 1,25(OH)_2_D/24,25(OH)_2_D ratio diagnosed important differences in their VitD status.

## Treatment

Based on Endocrine Society guidelines, all 3 patients received VitD3 supplementation (VitD capsules, 50,000 IU/week if body mass index (BMI) < 30 and twice a week if BMI ≥ 30 for 4 to 6 weeks followed by the maintenance dose of 1000 IU/day if BMI < 30 and 2000 IU/Day if BMI ≥ 30).

## Outcome and Follow-Up

VitD3 supplementation increased total levels of 25(OH)D from the range of 10.8 to 12.3 ng/mL (27-30.7 nmol/L) to the range of 31.7 to 33.8 ng/mL (79.1-84.4 nmol/L) and increased total levels of 24,25(OH)_2_D from the range of 0.5 to 0.9 ng/mL (1.3-2.3 nmol/L) to the range of 2.8 to 4.1 ng/mL (7-10.2 nmol/L). While PTH levels decreased from 62 to 41 pg/mL (6.6 to 4.4 pmol/L) in patient #1, PTH levels did not decrease in the other 2 patients. Because of adverse effects of hyperparathyroidism on bone, patient #1 would likely derive clinical benefit from suppressing PTH levels. Patient #3 appeared to be VitD sufficient and likely did not require VitD3 supplements for bone health. Patient #2 had subclinical VitD deficiency as reflected by suppression of 24-hydroxylase. In the absence of secondary hyperparathyroidism, it is controversial and outside the scope of this case report whether subclinical VitD deficiency has harmful effects or whether VitD3 supplementation would be beneficial.

## Discussion

Physiologists recognize that homeostatic regulatory mechanisms maintain healthy physiology [16, 17]. Endocrinologists leverage this notion to diagnose overproduction and underproduction of hormones (eg, measuring thyrotropin [TSH] to diagnose thyroid function) [18]. Endocrinologists apply the “free hormone hypothesis” to account for the impact of binding proteins on measured hormone levels. For example, pregnancy and estrogens increase thyroxine binding globulin levels thereby increasing total thyroxine levels [19]. Unfortunately, these concepts have not yet been incorporated into routine clinical assessment of VitD status. Because of inter-individual variation in VDBP levels, it is critical to account for this potential confounder [9, 14]. When treating VitD deficiency in association with secondary hyperparathyroidism, documentation of a decrease in PTH confirms that homeostatic mechanisms had been triggered prior to VitD3 supplementation. However, our mathematical model and the existing literature suggest that secondary hyperparathyroidism does not develop until VitD deficiency becomes relatively severe [2, 20, 21]. Nevertheless, even in milder VitD deficiency and in the absence of secondary hyperparathyroidism, the body triggers another homeostatic mechanism to maintain 1,25(OH)_2_D levels in the physiological range. Suppression of the 24-hydroxylase enzyme provides the first line of defense and is deployed at milder stages of VitD deficiency. This provides a rationale to incorporate 24,25(OH)_2_D measurement in assessing VitD status—especially, to differentiate patients with secondary hyperparathyroidism from patients with subclinical VitD deficiency but normal PTH levels. The 1,25(OH)_2_D/24,25(OH)_2_D ratio differentiated between patients #2 and #3 who had similar baseline total 25(OH)D levels. The 1,25(OH)_2_D/24,25(OH)_2_D ratio classified patient #2 as VitD insufficient and patient #3 as VitD sufficient. The use of VitD metabolite ratios, particularly the 1,25(OH)_2_D/24,25(OH)_2_D ratio, has 2 advantages over the traditional approach (total 25[OH]D measurement): accounting for VDBP levels and providing information about the body's response to VitD status as reflected by 24-hydroxylase activity. Notwithstanding the potential value of VitD metabolite ratios, we recognize the challenges in implementing a major change in diagnostic criteria—including, the need to assure standardization, harmonization, and quality control in the assays for 25(OH)D, 1,25(OH)_2_D, and especially the less widely available assay for 24,25(OH)_2_D.

## Learning Points

- Total plasma 25(OH)D does not account for the confounding effect of inter-individual variation in VDBP levels.
- VitD metabolite ratios provide VDBP-independent indices of VitD status.
- The body compensates for VitD deficiency by (a) suppression of 24-hydroxylation and (b) triggering secondary hyperparathyroidism.
- Ratios that incorporate 24,25(OH)_2_D levels reflect 24-hydroxylase activity.
- Preliminary evidence suggests that the 1,25(OH)_2_D/24,25(OH)_2_D ratio may provide an index that identifies patients most likely to exhibit secondary hyperparathyroidism.

## Data Availability

Primary data will be made available to qualified academic investigators for research purposes under a Data Transfer Agreement to protect research participants’ confidential information.

## Abbreviations

1,25(OH)2D: 1,25-dihydroxyvitamin D
24,25(OH)2D: 24,25-dihydroxyvitamin D
25(OH)D: 25-hydroxyvitamin D
BMI: body mass index
iPTH: intact parathyroid hormone
PTH: parathyroid hormone
VDBP: vitamin D binding protein
VitD: vitamin D
